# Design and fabrication of porous three‐dimensional Ag-doped reduced graphene oxide (3D Ag@rGO) composite for interfacial solar desalination

**DOI:** 10.1038/s41598-024-62987-z

**Published:** 2024-06-14

**Authors:** Fisseha A. Bezza, Samuel A. Iwarere, Hendrik G. Brink, Evans M. N. Chirwa

**Affiliations:** Water Utilization and Environmental Engineering Division, Department of Chemical Engineering, University of Pretoria, Pretoria, 0002 South Africa

**Keywords:** Interfacial desalination, Photothermal conversion, 3D graphene-based solar evaporator, Reduced graphene oxide, Solar-driven vapor generation, Environmental sciences, Energy science and technology, Engineering, Materials science

## Abstract

Solar-driven interfacial desalination technology has shown great promise in tackling the urgent global water scarcity crisis due to its ability to localize heat and its high solar-to-thermal energy conversion efficiency. For the realization of sustainable saline water desalination, the exploration of novel photothermal materials with higher water vapor generation and photothermal conversion efficiency is indispensable. In the current study, a novel 3D interconnected monolithic Ag-doped rGO network was synthesized for efficient photothermal application. The Ultraviolet–Visible-Near Infrared (UV–Vis-NIR) and FTIR analyses demonstrated that the controlled hydrothermal reduction of GO enabled the restoration of the conjugated sp^2^ bonded carbon network and the subsequent electrical and thermal conductivity through a significant reduction of oxygen-containing functional groups while maintaining the hydrophilicity of the composite photothermal material. In the solar simulated interfacial desalination study conducted using 3.5 wt.% saline water, the average surface temperatures of the 3D material increased from 27.1 to 54.7 °C in an hour, achieving an average net dark-excluded evaporation rate of 1.40 kg m^−2^ h^−1^ and a photothermal conversion efficiency of ~ 97.54% under 1 sun solar irradiance. In the outdoor real-world application test carried out, the surface temperature of the 3D solar evaporator reached up to 60 °C and achieved a net water evaporation rate of 1.50 kg m^−2^ h^−1^ under actual solar irradiation. The 3D interwoven porous hierarchical evaporator displayed no salt precipitation over the 54-h period monitored, demonstrating the promising salt rejection and real-world application potential for efficient desalination of saline water.

## Introduction

Water is vital for all forms of life, the core of socio-economic development, and the maintenance of ecological balance; hence, an adequate supply of freshwater is indispensable both in quality and quantity^1^. The demand for freshwater is ever-increasing, with rising population size, industrialization, and urbanization demanding the supply of enough water both in quality and quantity. Despite its abundance, covering about 3/4th of the earth’s surface, freshwater is a scarce resource, as more than 97.5% is salty water placed in oceans and seas, and only the remaining 2.5% is found as freshwater that exists in rivers, lakes, groundwater, and polar ice caps^2^. To address the freshwater scarcity, new and innovative approaches should be explored to generate new water resources and exploit the untapped immense and inexhaustive saline water in oceans and seas that is not suitable for use^1^. Desalination is the most sustainable option in tackling the pressing issue of rising water scarcity worldwide by harnessing the vast and untapped seawater resource^3^. However, the current thermal and membrane-based desalination methods, including reverse osmosis, electrodialysis, multiple-stage flash distillation, and electrodialysis, are energy-intensive and unaffordable^3^.

Solar desalination technology solely relying on inexhaustible solar energy is the most sustainable technology for tackling the global freshwater scarcity in many parts of the world. However, traditional solar-driven desalination technologies have low efficiency, as low as 30–45%, due to bottom or volumetric heating prone to radiative, convective, and conductive heat losses, requiring more surface area and additional cost, limiting their practical application^4,5^. Recently, solar-driven interfacial evaporation technology has been gaining increasing attention owing to its high photothermal conversion efficiency, reaching up to 99% due to the surface localization of heat harvested and vapor generation at the air–water interface. Efficient utilization of heat harvested would be realized through thermal insulation of the absorber by avoiding direct contact with the bulk water to suppress heat loss to the bulk water^5^ This strategy further favors attaining energy efficiency even more than theoretical energy efficiency via recycling the latent heat and reducing the enthalpy of evaporation.

Through the design of efficient solar absorbers that harvest solar energy and efficient thermal management, the solar energy harvested is converted to heat and localized on the air–water interface to generate vapor, which is condensed to freshwater^5,6^. A solar-driven interfacial desalination system encompasses three essential components: (1) efficient photothermal material with broadband solar absorption potential over the entire solar spectrum and solar to thermal conversion potential; (2) an efficient thermal insulation system to avoid heat loss to the bulk water; and (3) water transfer channels that continuously supply adequate water to the evaporative surface^7^. The solar absorber (photothermal material) is the core component of the interfacial desalination system, and an ideal photothermal material has outstanding broadband solar absorption, high photothermal conversion efficiency, ideal porosity, and scalability. An ideal photothermal material should have excellent broadband light absorption performance over the entire spectrum ranging from 200 to 2500 nm to ensure efficient photothermal performance^8^. Efficient broadband optical absorption, photothermal conversion efficiency, ideal porosity, and scalability are the key factors for the design and construction of novel photothermal materials or reengineering of the former solar absorbers^9^. Photothermal materials can be broadly classified as plasmonic metals, semiconductors, carbonaceous materials, and polymers^10^*.* Plasmonic materials like Ag, Au, and Cu are efficient photothermal materials as a result of the localized surface plasmon resonance (LSPR) phenomena, which occur when the incident light frequency aligns with the electrons’ frequency on the material's surface, there is a notable increase in photothermal conversion efficiency^6^^11^. Despite having efficient photothermal conversion efficiency, plasmonic metals are responsive over a limited spectral range, which is below optimal for solar energy harvesting; this limited plasmonic response can be broadened by tuning the size, shape, and assembly configuration of the NPs, hybridization with other plasmonic nanoparticles, or constructing composites with other photothermal material^5,12^. Novel metal nanoparticles like Ag NPs and Au NPs have been explored for solar energy conversion applications owing to their plasmonic response in the broadband solar UV visible and near IR regions, following shape, size, and assembly configuration, which are well matched with the solar spectrum^13^. Carbonaceous materials like carbon dots, black carbon, graphite, graphene, reduced graphene oxide activated carbon, carbon soot, and carbon nanotubes have demonstrated efficient photothermal performance owing to their broadband absorption over the entire solar spectra owing to the p band’s excitation, porous structure, low cost, and chemical stability^14,15^.

Despite the continuous design and development of state-of-the-art photothermal materials, the increase of solar-driven water evaporation rate via improving the solar–thermal energy conversion has almost reached its theoretical upper limit^16,17^. Alternatively, the current research focus is on enhancing the evaporation rate through design and development of innovative structures and configurations to boost vapor generation performance. Specifically, the construction of three-dimensional (3D) photothermal architectures with large surface area is demonstrating a promising potential^17,18^. In contrast to traditional 2D solar interfacial desalination systems, which suffer from increased conductive heat loss due to full exposure to sunlight, 3D evaporators have additional surfaces that are protected from incoming solar radiation. These surfaces maintain lower temperatures than the surrounding environment and can utilize alternative environmental energy sources such as ambient temperature and convective flow to enhance the rate of evaporation^19,20^. Different types of 3D evaporators, including spiral, cylindrical cup-shaped structures, and funnel-shaped structures, have been created. These innovative designs are capable of efficiently transferring water, capturing diffuse light energy, and minimizing energy losses. Consequently, they are capable of efficient utilization of solar energy and other environmental energy sources, leading to an impressive photothermal energy efficiency beyond 100%^21^. By employing rational design and advanced thermal management systems, significant improvements in water evaporation performance have been achieved through the development of various 3D solar evaporators^17,18,22,23^. Wu et al.^23^ employed 3D printing technology to create a distinctive three-dimensional bionic conical evaporation system. This system showcases a water evaporation rate of 2.28 kg m^−2^ h^−1^, surpassing that of 2D interfacial solar evaporation systems (1.07 kg m^−2^ h^−1^), thereby demonstrating the practical potential of the three-dimensional integral conical evaporator in seawater desalination. In a related study, Wu et al.^24^ developed a 6-fin photothermal heatsink-like evaporator (HSE), which achieves an impressive evaporation rate of 4.10 kg m^−2^ h^−1^, equivalent to an energy conversion efficiency of 170% of the received solar energy.

Graphene is a single-atom-thick, 2D sheet of sp^2^ hybridized carbon atoms with extraordinary physicochemical properties, like high mechanical strength, a high specific surface area of up to 2630 m^2^ g^−1^, high electrical conductivity (10^8^ A cm^−2^), high thermal conductivity (∼2000 to 5000 W m K^−1^), having a great potential application for solar energy storage, solar energy harvesting, photocatalytic applications, etc.^25^. Yet the restacking or aggregation of graphene nanoplates due to π–π interactions or a deficiency of interconnection severely limits its application at the macroscopic level. Conversion of the 2D graphene sheets into a 3D graphene monolithic structure has been documented as an ideal approach to exploiting the full potential of the graphene at the macroscale while maintaining the intrinsic properties of graphene at the nanoscale level^26^. In addition to preventing aggregation of graphene nanosheets, 3D graphene-based macrostructures exhibit multiple functionalities, like high specific surface area, lightweight, covalently interconnected hierarchical network of sp^2^ hybridized carbon network providing an excellent highway for the transport of electrons, photons, and electron transfer with a tuneable band gap, high porosity and microcavities/channels for mass, as well as hydrophilicity for easy water transport to the surface^25,26^.

The mesoporous 3D graphene-based structures offer numerous nanoscale advantages in practical applications, including an increased evaporation surface area that provides a greater number of evaporative interfaces per unit area^27^. Reduced graphene oxide, produced through the oxidation and subsequent reduction of graphene oxide, exhibits physicochemical properties similar to graphene, such as high electrical conductivity, specific surface area, stable mechanical strength, and exceptional light absorption potential across the entire broadband solar spectrum (250–2500 nm)^3^. Moreover, the incorporation of plasmonic nanoparticles (NPs) into the 3D graphene porous structure significantly enhances its photothermal performance, owing to the synergistic effect between the localized surface plasmon resonance of the AgNPs and the broadband solar absorption capability of graphene material^13,28^. The design and synthesis of hybrid materials combining graphene or graphene derivatives with metallic, semiconductor or plasmonic nanoparticles offers a range of enhanced physicochemical properties that overcome the limitations of each individual material categories^6^. By incorporating carbon-based materials like carbon black (CB), graphite, and reduced GO (rGO) with semiconductors or plasmonic nanoparticles, the concentration of free charge carriers (electrons or holes) is significantly increased throughout the photon absorption layer, leading to the simultaneous activation of thermal vibration across the semiconductor lattice and enhances excitation among the π orbitals in the reduced rGO. As a result, the solar to heat conversion process is maximized through synergistic photothermal effects^6,11^. Various three-dimensional monolithic rGO/metallic, semiconductors or plasmonic material nanocomposites, such as rGO/Ag, MnO_2_/C, rGO/CuS, MoS_2_/C, MnO_2_/C, and CuO/C composites have been proven to exhibit exceptional steam generation and desalination capabilities^17,19,20,29–32^. Kospa et al.^30^ achieved an impressive evaporation rate and solar-to-steam efficiency using a CuO-rGO/PANI composite evaporator, showcasing high salt-resistance and oil-repulsion properties. Xiao et al.^31^ developed a multi-level 3D rGO@Ag hybrid photothermal material with exceptional photothermal performance. Under one-sun irradiation, this material achieved a freshwater generation rate of 1.56 kg m^−2^ h^−1^, with a remarkable photothermal conversion efficiency of 97.9%. Kang et al.^32^ developed an efficient silver-loaded graphene (GO-Ag) and polyvinyl alcohol/chitosan (PVA/CS) composite that exhibited up to 97% solar absorption performance, achieving an evaporation rate of 1.64 kg m^−2^ h^−1^ under one-sun conditions, accompanied by an outstanding photothermal conversion efficiency of 99.99%.

Currently, the main challenge facing the interfacial 3D evaporators is the issue of scaling and salt crystal formation on the evaporator surface, which negatively impacts solar absorption, blocks water transfer channels, adversely affects photothermal conversion efficiency, leading to interruptions in the interfacial desalination process and compromising long-term stable performance^17,19,20^. Therefore, the current research hotspot lies in the design and development of efficient self-cleaning and salt-resistant 3D hybrid photothermal materials that can deliver stable performance, high photothermal conversion rates, and effective water vapor generation^17,32–34^. Consequently, there is a need to develop new solar steam generation devices that possess adequate salt-resistance capabilities, efficient solar-thermal energy conversion, and high-water evaporation efficiency. This study presents a robust 3D solar-driven interfacial evaporator that exhibits exceptional salt rejection, self-cleaning properties, high photothermal conversion efficiency, and vapor generation potential, ensuring stable performance over an extended period through the incorporation of Ag into the 3D rGO monolithic structure.

## Materials and methods

### Materials

Graphite, ortho-phosphoric acid (H_3_PO_4_), sulphuric acid (H_2_SO_4_), H_2_O_2_, silver nitrate (AgNO_3_), sodium borohydride (NaBH_4_), potassium permanganate (KMnO_4_, 99.9%), and hydrogen peroxide (H_2_O_2_, 30%), hydrochloric acid (HCl, 30%) were purchased from Sigma-Aldrich (St Louis, MO).

### Synthesis of Ag-doped 3D reduced graphene oxide (3D Ag@rGO)

#### Synthesis of GO

Graphene oxide was synthesized using Tour's method, as previously described by Bezza et al.^35^. Briefly, 360 mL of sulphuric acid (H_2_SO_4_) and 40 mL of ortho-phosphoric acid (H_3_PO_4_) were combined in a ratio of 9:1 in a 2 L Erlenmeyer flask. The mixture was then stirred using a magnetic stirrer for 15 min. Following this, 3.0 g of graphite and 18 g of KMnO_4_ were gently added to the stirring solution, and the stirring process was continued for 1 h, gradually increasing the temperature to 40–45 °C. The Erlenmeyer flask was transferred to an orbital shaker and shaken for a period of 24 h at a temperature of 50 °C and a speed of 200 rpm. To stop the oxidation reaction, 400 mL of ice water and 30 mL of 30 wt.% H_2_O_2_ were added. The resulting graphene oxide (GO) was then collected through centrifugation for 10 min at a speed of 8000 rpm. To ensure purity, the collected GO was washed repeatedly with 200 mL of HCl (30% wt.%, 200 mL).

#### Synthesis of 3D Ag@rGO

**Figure 1:**
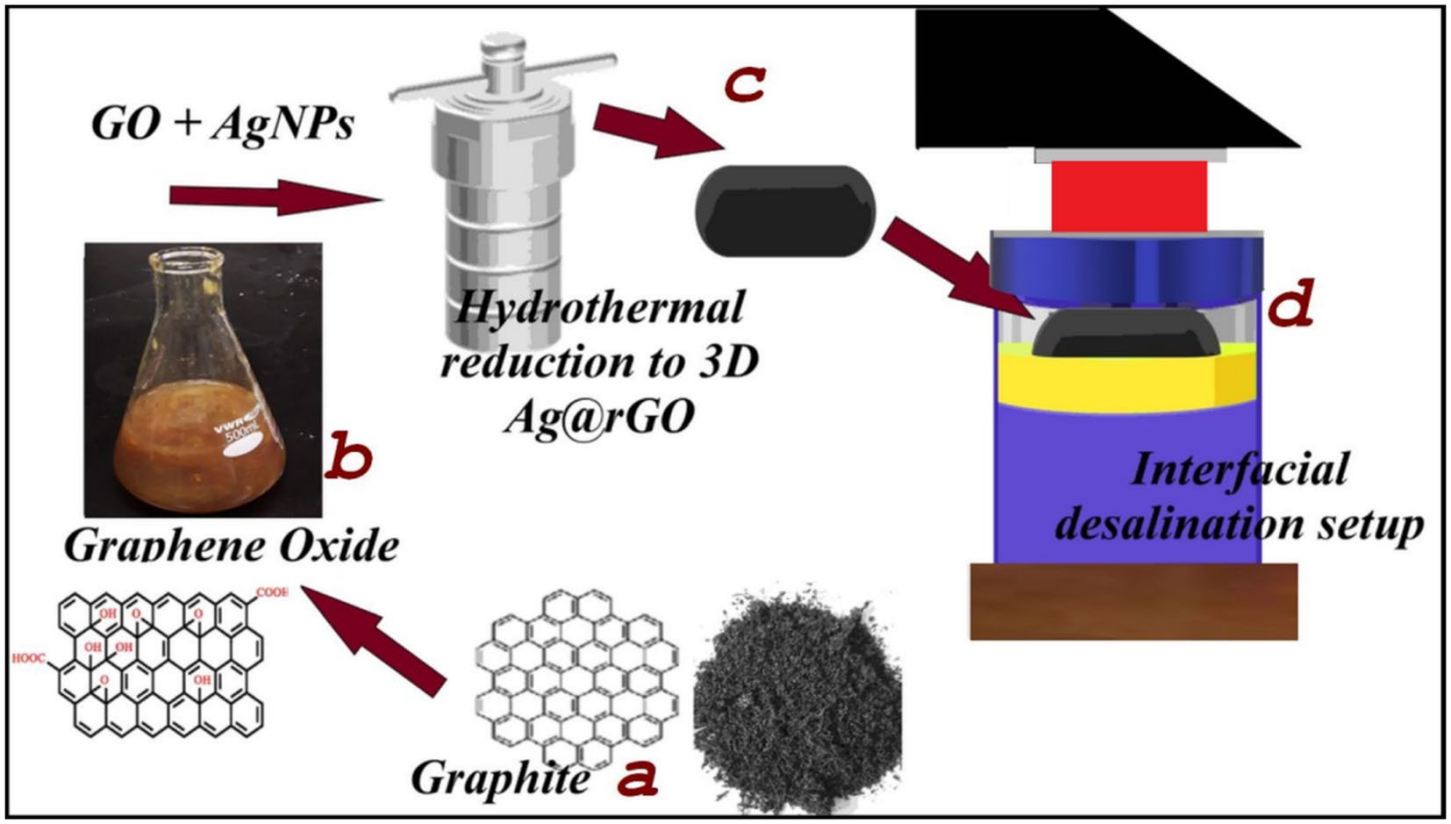
Schematic illustration of hydrothermal reductive synthesis of 3D Ag@rGO from graphene oxide (GO). GO is obtained from the oxidation of graphite nanosheets through Tour’s method, and used for the 3D monolith synthesis, which is placed in the interfacial desalination setup.

The synthesis method involves the combination of two well-established and facile methods: a one-step citrate stabilized Ag nanoparticles synthesis by reduction of AgNO_3_ using NaBH_4_ as previously described by Haber and Sokolov Haber^36^. The rGO is a chemically modified graphene with a controlled reduction of oxygen-containing functional groups and exhibits graphene-like physicochemical properties, including thermal and electrical conductivity and broadband solar light absorption potential^37^. 3D Ag-doped rGO was synthesized via hydrothermal reduction of 300 mL GO/AgNPs aqueous solution, consisting of 10 mg mL^−1^ GO and 5 mg mL^−1^ of AgNPs. The mixture was vigorously mixed and ultrasonicated for 60 min. The resulting sample was then transferred to a 300 mL Teflon-lined autoclave and heated at a temperature of 180 °C for 24 h. Afterwards, the 3D product was gently taken out and freeze-dried for 72 h. Subsequently, the 3D Ag-doped rGO hydrogel obtained through one-step hydrothermal reduction of GO solution in the presence of the AgNPs as depicted in Fig. 1 was subjected to annealing and employed for the solar driven interfacial desalination study.

### Characterization

The microstructure and elemental composition of the Ag-doped rGO sample were characterized using field emission scanning electron microscopy (FESEM) coupled with energy-dispersive X-ray (EDX) analysis (EDS, JEOL-7800F). The UV–Vis-NIR spectrophotometer (UV3600, Shimadzu Scientific Instruments), was employed to evaluate the broadband solar absorption potential of the 3D Ag@rGO absorber across the entire solar spectra range (250–2500 nm) as previously described^35^. X-ray diffraction (XRD) analysis was conducted to explore the crystallinity and phase purity of the sample produced with Cu-Kα radiation (λ = 1.540 Å) in the 2θ range from 5 to 90° at a step size of 0.05°/s. Fourier transform infrared (FTIR) analysis was conducted to determine the functional groups present in the samples. A FTIR Perkin Elmer 3100 spectrometer was utilized, with a spectral range of 4000–400 cm^−1^, an increase of 32 scans, and a resolution of 0.2 cm^−1^.

### Solar driven interfacial desalination performance test

**Figure 2:**
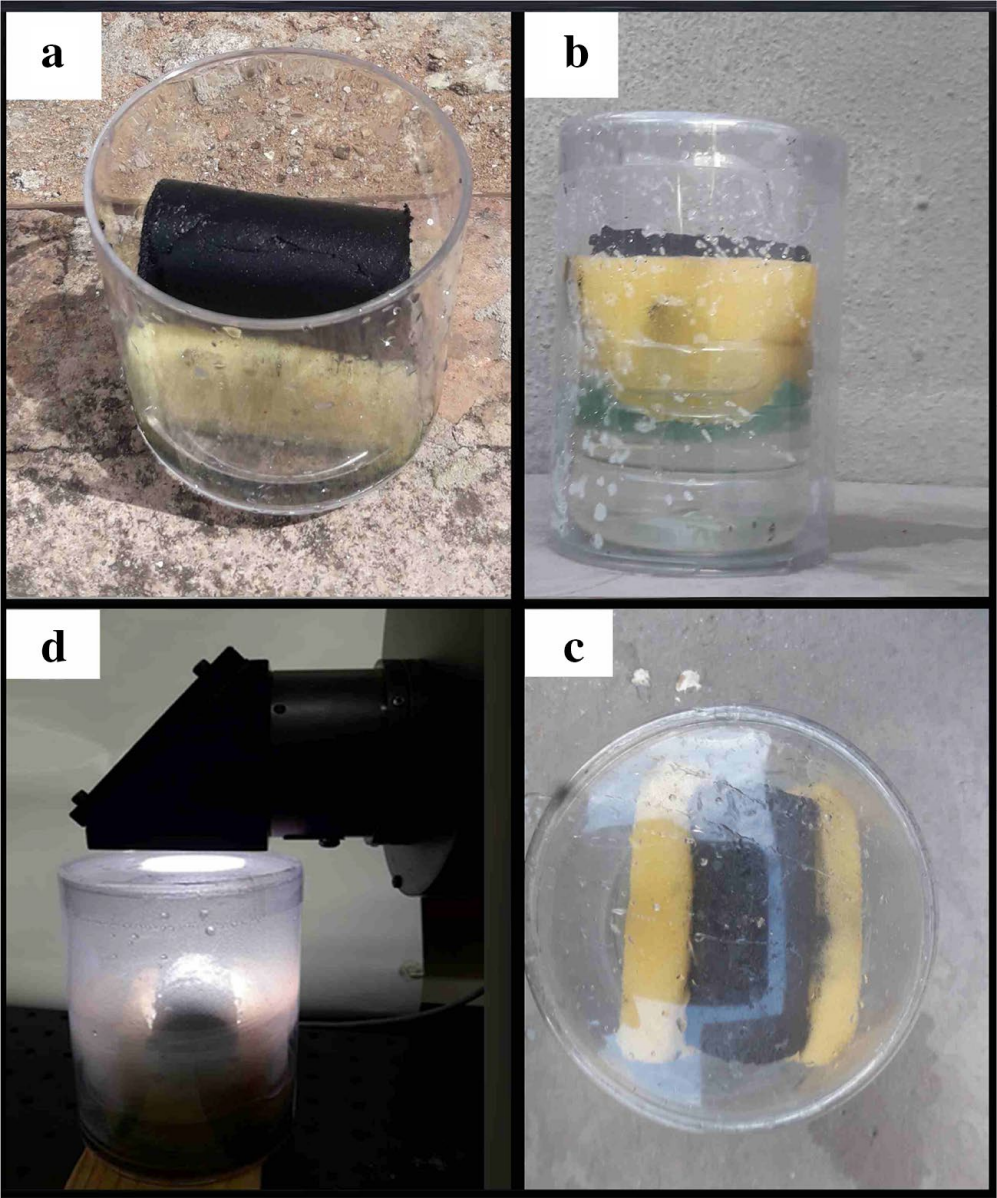
Digital photographs of 3D Ag@rGO evaporator-based interfacial desalination set up with the 3D Ag@rGO photothermal aerogel placed on a thermally insulated and hydrophilic cellulose sponge that maximizes solar utilization as well as facilitates the fast transfer of water.

The solar-driven interfacial desalination study was conducted using a solar simulator, which is based on a xenon-arc lamp (SS-X200R), under 1 sun illumination, which is equivalent to 1 kW m^–2^. To prevent conductive heat loss to the bulk water and facilitate fast water transfer to the evaporative surface, a 3D Ag@rGO aerogel measuring 7.0 cm × 3.0 cm was placed on a cellulose sponge. The cellulose sponge, with a low thermal conductivity of 0.043 W m^−1^ K^−1^, was in turn placed in a small vessel containing saline water. To minimize convective and radiative heat losses and localize the generated heat on the evaporative surface, the vessel was enclosed in a glass with a transparent polymeric lid as illustrated in Fig. 2. The evaporation rate was determined by measuring the mass change of the working fluid in the beaker using an analytical balance (Analytical Balance MS205DU). In order to simulate seawater, water with a salinity of 3.5 wt.% was used in the desalination test^27^.

Figure 2 displays the cylindrical 3D Ag@rGO structure placed on a cellulose sponge and immersed in saline water in a vessel (a). The interfacial desalination setup is then enclosed by a transparent polymeric lid, as seen in the side view (b) and top view (c). The setup is placed under a solar simulator (d) and the surface temperature of the absorber is monitored using an infrared (IR) camera (TG165, FLIR Systems Inc.). To evaluate the performance and long-term stability of the solar absorber’s interfacial desalination performance, two separate tests were conducted, each lasting 27 h, resulting in a total of 54 h. Each test consisted of three rounds, with each round lasting nine hours. Furthermore, the interfacial desalination setup was also evaluated for its potential to generate freshwater in real-world outdoor conditions by placing it on a rooftop and exposing it to natural sunlight irradiation.

The evaporation rate, with units of kg m^─2^ h^─1^, is determined using Eq. (1)^8^.

1 $$\dot{m}\;{ = }\;\frac{\Delta m}{{S \times t}}$$

where ∆m is the change in mass of the water in the vessel accounted for as vapor generated (kg), S is the effective solar absorption area (m^2^), which is the top cylindrical area of the 3D evaporator that is directly exposed to the solar irradiation (32.76 cm^2^), and t refers to the solar irradiation time (h).

The photothermal conversion efficiency ($\eta$), which is the percentage of the solar irradiance that has been stored in the vapour generated to the incoming solar flux, is determined using Eq. (2)^8,38^.

2 $$\eta = \frac{{ \dot{m} \cdot \left( {L_{v} + Q} \right)}}{{P_{i} }}$$

3 $${\text{Q}}\, = \,{\text{C}}\Delta {\text{T}}$$

where ${\dot{\text{m}}}$ represents evaporation rate of the generator following the deduction of the evaporation rate in a dark field, in kg m^─2^ h^─1^, ${\text{L}}_{{\text{v}}}$ enthalpy of vaporization (kJ/kg) and Q signifies sensible heat in increasing surface temperature from T_1_ to T_2_ and P_i_ represents the standard solar illuminance (1 kW m^─2^). C is the specific heat capacity of saline water (3.993 J g^−1^ K^−1^), ∆T represents the change in surface temperature of water. An inductively coupled plasma optical emission spectroscopy (ICP-OES) analysis was performed using inductively coupled plasma optical emission spectroscopy (PerkinElmer Optima 8300 ICP-OES) to measure the primary salt ion concentrations before and after the desalination test, in order to evaluate the quality of the desalinated water for potable use.

## Results and discussion

### Structural and morphological characterization

**Figure 3:**
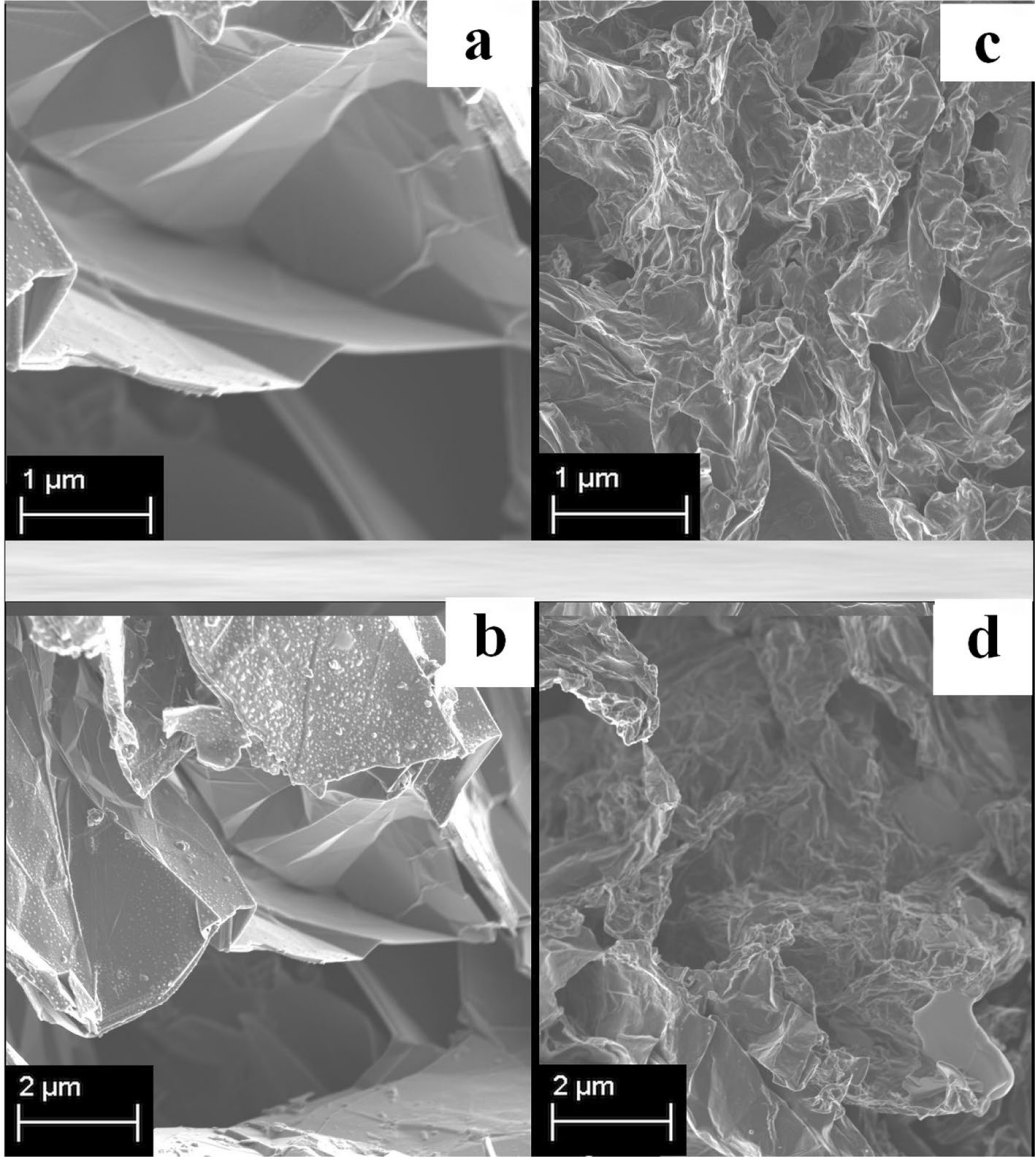
FESEM images of (**a**, **b**) pristine graphene and (**c**, **d**) silver nanoparticle (AgNPs)-doped rGO at different scales.

After the formation of the 3D rGO hydrogel doped with Ag nanoparticles, it is subjected to freeze-drying in order to maintain its interconnected porous structure in three dimensions. The resulting hierarchical 3D monolith, composed of reduced graphene oxide layers that are covalently bonded, showcases physicochemical properties reminiscent of graphene, including remarkable conductivity, exceptionally low weight, extensive surface area, and impressive mechanical stability^25^. As displayed in Fig. 3c and d, in the FE-SEM images, the 3D Ag@rGO composite exhibited highly porous, wrinkled surface morphology, and micropores indiscriminately spread over the porous surface compared to the pristine graphene (Fig. 3a, b), demonstrating the successful preparation as intended.

**Figure 4:**
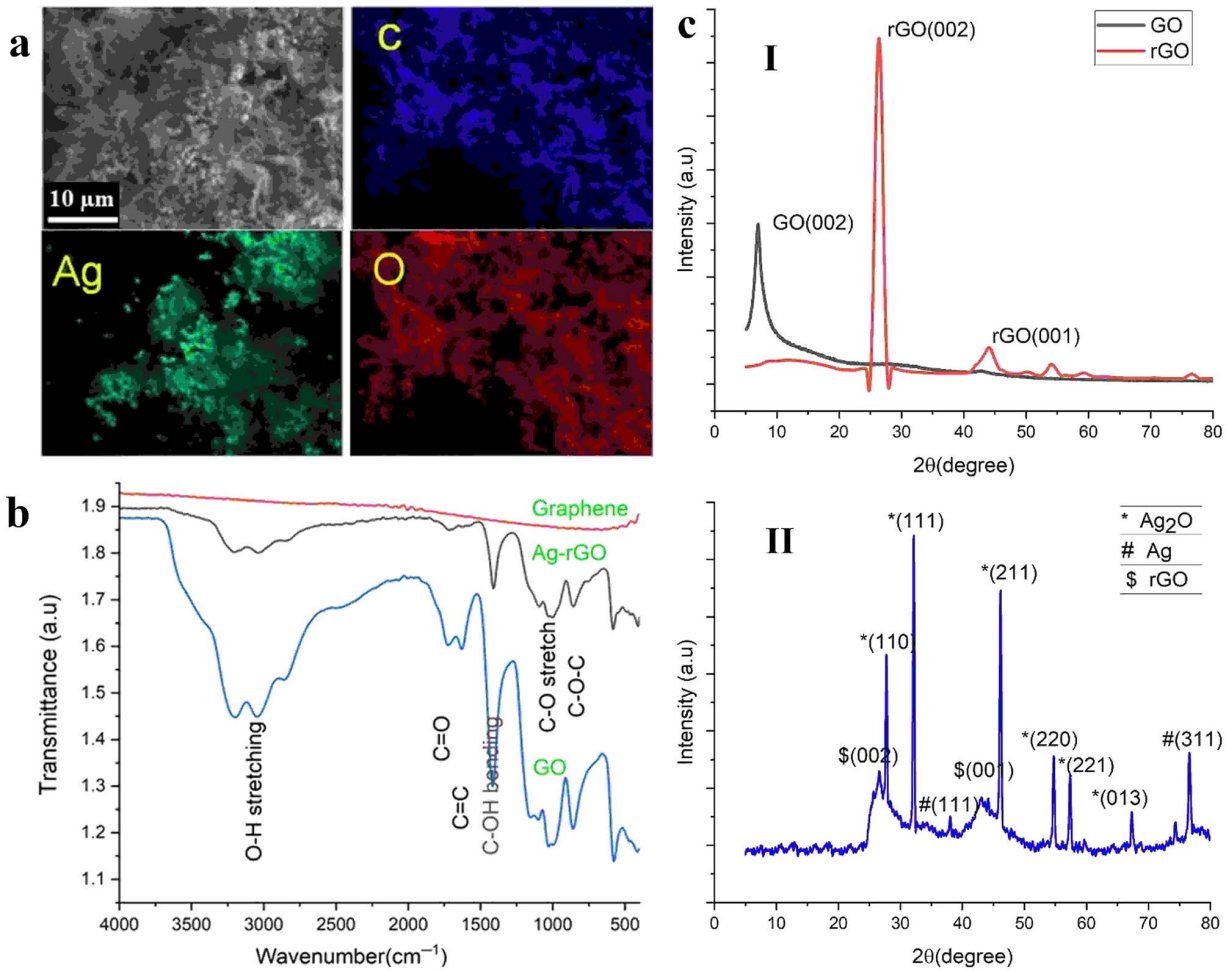
EDX elemental mapping of the Ag-rGO photothermal material exhibiting uniform dispersion of the Ag dopant in the sample (**a**); FTIR spectra of GO (blue line), Ag@rGO (black line), and pristine graphene (red line) (**b**); XRD patterns (**c**) of GO, rGO (I), and Ag@rGO (II).

An in-depth examination of the field-emission scanning electron microscopy (FESEM) images of the specimens (Fig. 3) demonstrates that, although both the pristine graphene and Ag@rGO possess porous microstructures characterized by numerous wrinkled and fluffy features, the Ag@rGO sample displays an exceptionally high degree of wrinkling and defectiveness. The imperfections and irregularities observed in the reduced graphene oxide (rGO) are attributed to the presence of oxygen-containing functional groups on the surfaces and edges of the rGO nanosheets^39^. The high porosity, abundant microcavities, pores, and fluffy sponge-like structure of the 3D Ag@rGO would effectively facilitate mass transfer^40^. EDX elemental mapping of the Ag@rGO nanosheet (Fig. 4a) shows that the AgNPs are uniformly dispersed on the porous structure of the rGO sheets.

Graphene oxide (GO) is an oxidized graphite having oxygen-containing functional groups like epoxy functional groups and carbonyl functional groups intercalated on its basal planes and edges^41^. GO is comprised of sp^3^ hybridized domains covalently bonded to the oxygen-containing functional groups, disrupting the sp^2^ domains at the basal plane and edges of graphene, and some sp^2^ conjugated domains having mobile π electrons^41,42^. Graphene oxide is an electrical hybrid containing the conductive π states of sp^2^ hybridized carbon networks and the large energy gap (band gap) between the σ states of sp^3^ hybridized carbon atoms. GO is thermally insulated and weakly conductive due to the disruption of sp^2^-conjugated carbon bonds by sp^3^ oxygen functional groups containing domains^43^. The electrical conductivity can be restored through reduction of the sp^3^ conjugated domains in σ states, through the removal of oxygen-containing functional groups, and by increasing sp^2^ conjugated carbon networks and π electron conjugation^44^. The large band gap of GO can be reengineered or adjusted using the incomplete removal of oxygen-containing functional groups through reduction^43^. After the ultrasonic combination of the Ag NPs with the GO dispersion, subsequent hydrothermal reduction of the GO to rGO, and freeze drying, a hierarchical 3D rGO architecture was obtained (Fig. 2). Fourier transform infrared (FTIR) spectroscopy was conducted to verify the formation of GO and evaluate the degree of reduction of the Ag-rGO samples (Fig. 4b).

The GO exhibited strong adsorption peaks at 1734 cm^−1^ corresponding to C=O stretching vibration from carbonyl and carboxylic groups^45^, 1634 cm^−1^ ascribed to the aromatic C=C stretching or O–H bending vibration, 1416 cm^−1^ (O–H, –OCH bending)^46^, 1029 cm^–1^ owing to the C–O stretch of alkoxy group, and the broader peak around 3200 cm^−1^ represents hydroxyl *C*–OH functional groups, respectively^45,47^. As shown in Fig. 4b, upon thermal reduction, the Ag-rGO demonstrated a significant reduction of the oxygen-containing functional groups, predominantly hydroxy-OH (3594 cm^−1^), carbonyl (1730 cm^−1^) and –OH (1418 cm^−1^), compared to GO, consistent with previous reports^41,48^. Through the controlled reduction of GO, the amount of oxygen-containing functional groups and the sp^3^ carbon network can be significantly removed and replaced with the sp^2^ conjugated carbon networks, generating graphene-like rGO with remarkable electrical conductivity for the continuous electron pathway^49^. The degree of reduction of the rGO can be monitored by modulating the amount of oxygen-containing functional groups as well as the time of hydrothermal reduction (rGO)^50^.

X-ray diffraction (XRD) analysis was conducted to evaluate the structural properties and phase information of the reduced graphene oxide composite. As presented in Fig. 4c, I, the XRD diffractogram of GO displayed a strong diffraction peak at 2θ = 9°, which is ascribed to the (002) plane of graphene oxide sheets, with a corresponding interlayer distance of 0.98 nm, according to Bragg’s law^50,51^. Up on thermal reduction, the 002-plane shifted to 2θ = 25.4° with a corresponding interlayer distance of ∼ 0.350 nm. An additional weak peak of rGO is observed at 43°, which corresponds to the [001] plane of graphene^51^. The higher interlayer distance between the two graphene oxide layers displays the intercalation of the oxygen-containing functional groups; the significant reduction in the interlayer distance from ∼ 0.98 to ∼ 0.350 nm subsequent to the thermal reduction of GO indicates the elimination of the oxygen-containing functional groups, decreasing the band gap, and restoring the sp^2^ hybridized carbon domains of the graphene-like rGO structure^52^. Thermal reduction of GO significantly removes the oxygen-containing functional groups, increases π–π interactions, and restores the in-plane sp^2^ hybridized carbon structure of the modified graphene sheets^53^. The incorporation of the Ag_2_O NPs on rGO nanosheets was confirmed by XRD analysis. As shown in Fig. 4c, II, the prominent peaks observed at 2θ values of 27.71°, 32.15°, 46.11°, 54.76°, 57.21°, 67.3° represent the (110), (111), (211) (220), and (222) planes assigned to the cubic phase of Ag_2_O (JCPDS file No. 76–1393)^54,55^.

### Solar absorption over Ultraviolet visible near infrared (UV–Vis–NIR) absorption spectra

**Figure 5:**
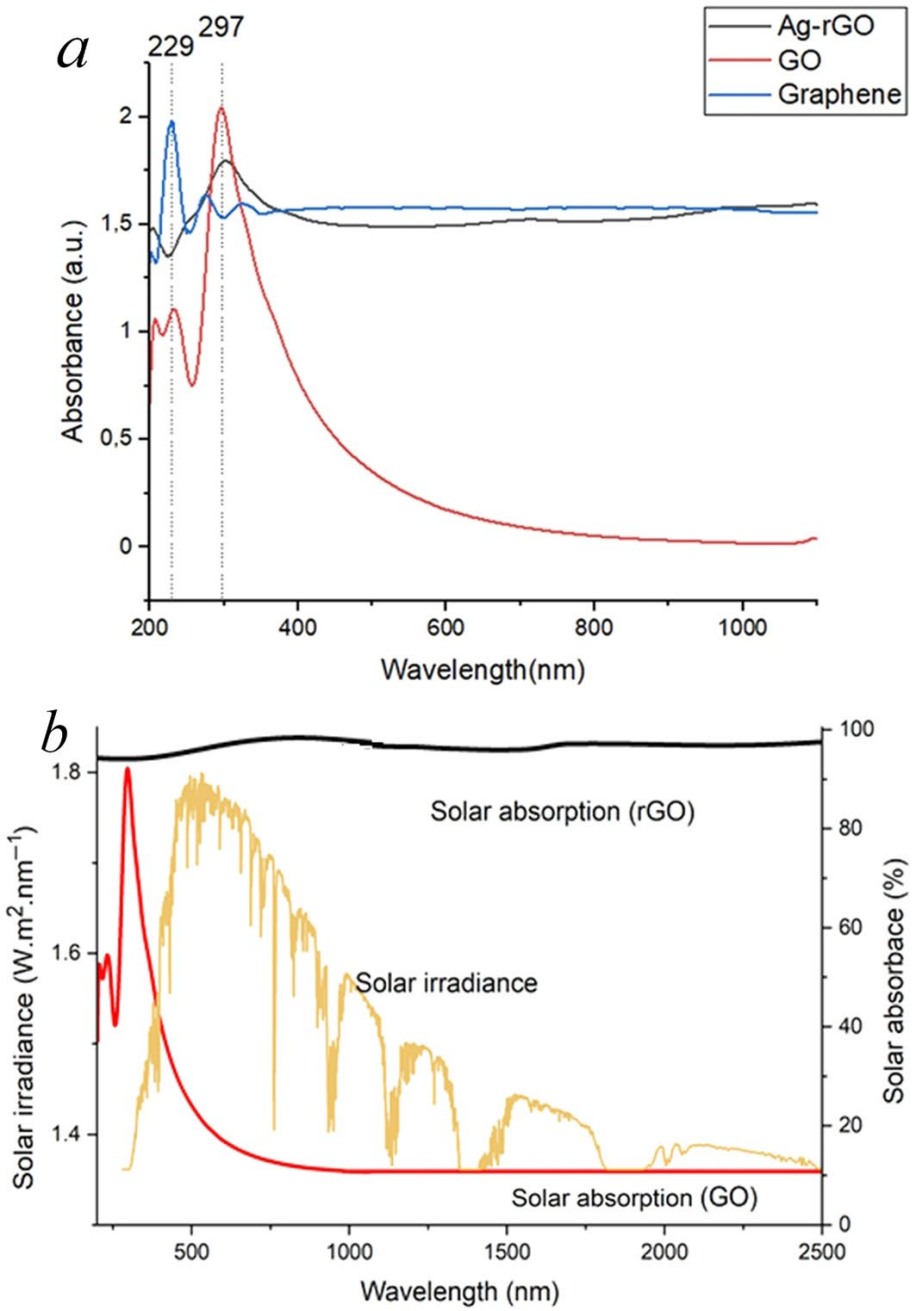
Optical property (absorption intensity) of the fabricated rGO (brown) and GO (red) compared to pristine graphene in the UV–Vis-NIR broadband region monitored (200 − 1100 nm) (**a**): percentage absorption performance of the 3D Ag@rGO and GO in the UV–Vis-NIR broadband solar irradiance (200 − 2500 nm) (**b**).

To realize high-efficiency desalination, the solar absorber needs to have high solar absorption in the broadband UV–Vis-NIR region and strong capillary wicking capability in the porous hierarchical structure^56^. The light-to-heat conversion performance of the photothermal material depends on its light absorption potential^3,53^. As shown in Fig. 5a, b, the 3D Ag@rGO exhibited remarkable light absorption performance across the UV–Vis-NIR regions (200–2500 nm). In comparison, it can be observed that the GO showed very low light absorption over the entire UV–Vis range owing to its low electrical conductivity due to the breakup of the sp^2^ conjugated carbon network of graphene^41^.

After hydrothermal reduction, the π-conjugation and, consequently, the electrical conductivity of the rGO were restored, and Ag@rGO exhibited robust solar absorption performance in the entire UV–Vis-NIR range, as displayed in Fig. 5a and b^49^. The restoration of the conjugated π bond structures in rGO enhances the absorption of photons in the NIR region and activates its orbital electrons, giving the Ag@rGO remarkable photothermal performance^57,58^. Moreover, the high UV–Vis-NIR broadband solar absorption performance observed is attributed to the synergistic free electron-induced LSPR effect of Ag NPs and the reduced graphene oxide (rGO) electrical conductivity^57,59^.

Figure 5a presents the UV–Vis–NIR absorption spectra of GO, Ag-rGO, and pristine graphene following the absorption of electromagnetic radiation. The GO displays peaks at 229 nm representing the transition from π → π* of C═C aromatic bonding, and the peak at 297 nm designates the n → π* transition of C═O bonds^60,61^. Subsequent to GO reduction, the π–π* peak at 229 nm red-shifts to 297 nm; on the other hand, the peak at 306 nm completely disappears, demonstrating the significant removal of the oxygen-containing functional groups, the generation of graphene-like rGO, and the restoration of the sp^2^ conjugated carbon network with significantly increased broadband absorption of the UV–Vis-NIR entire solar spectra to an extent comparable to the pristine graphene^61,62^.

**Figure 6:**
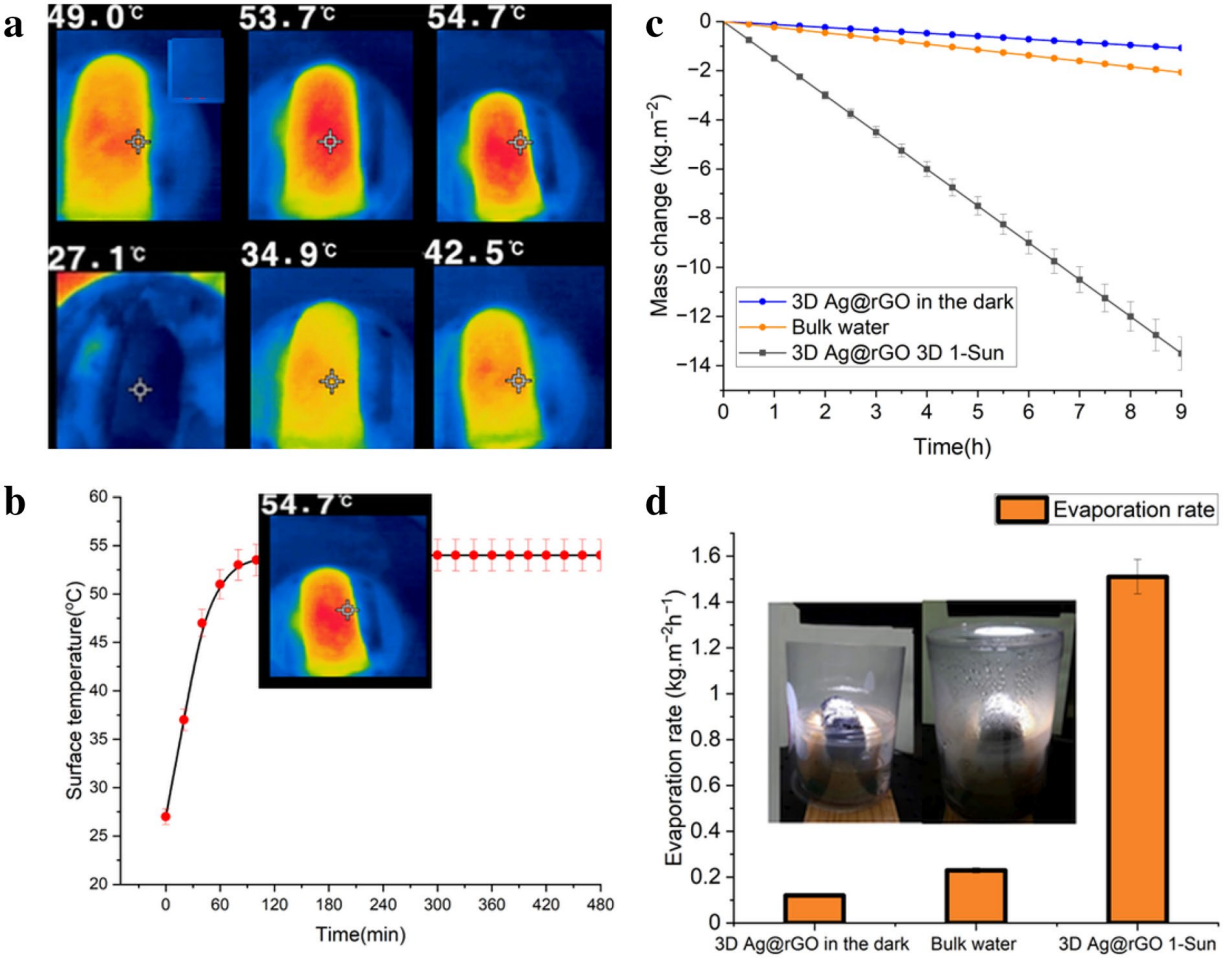
Average surface temperature response profile of the 3D Ag@rGO (**a**); IR thermal images of the 3D Ag@rGO, under 1 sun solar irradiation captured every 20 min from an initial surface temperature of 27.1 °C to ~ 54.7 °C over an hour (**b**); stable solar-driven interfacial desalination performance of the 3D Ag@rGO with a significant change in the mass of the 3.5 wt.% saline water over a period of 27 h in a study conducted in three rounds, each lasting nine hours compared to a reference run where direct bulk water heating was used without the evaporator (**c**); and average evaporation rate of 3.5 wt.% saline water measured over the same 27-h duration under dark conditions, direct bulk heating, and 1 sun irradiation (**d**); a photograph illustrating the experimental setup of solar-driven interfacial evaporation was included, which depicted the increasing water vapor generation over time under 1 sun solar irradiation (*inset*).

As observed in Fig. 5b, the reduced rGO demonstrated remarkable broadband solar absorption potential (up to ~ 98%) over the broadband UV–Vis–NIR range. The possibility of regulating the optical properties of GO-based monolithic structures through thermal reduction makes them ideal candidates for efficient photothermal performance^61,62^. The hydrothermal reduction of GO to rGO increases the solar absorption efficiency as the disrupted sp^2^ bonding orbitals of graphene are restored, increasing electrical conductivity. Thus, controlled thermal reduction restores the electrical conductivity of GO to a degree close to pristine graphene by tuning the oxygen content in the rGO^44,61^. In addition to high electron mobility and electrical conductivity, the rGO defective sites left after the elimination of oxygen-containing functional groups are chemically active and play a critical role in enhancing the photothermal performance of the reduced graphene oxide^63,64^. The photothermal performance of the 3D Ag@rGO was examined by continuously monitoring its surface temperature using an infrared camera while exposed to simulated solar irradiation of 1 kW m^−2^. In Fig. 6a, the IR thermal images of the 3D Ag@rGO reveal a temperature increase from 27.1 °C to a steady state temperature of ~ 54.7 °C within 60 min under 1-Sun solar irradiation, indicating excellent photothermal performance. Figure 6b illustrates the average surface temperature response profile of the 3D Ag@rGO evaporator.

### Desalination performance

The solar-driven interfacial desalination performance of the solar absorber was investigated both indoors using a solar simulator and outdoors under natural environment in intense sunlight with an average ambient temperature of ~ 32 °C. Figure 1c illustrates the concept of thermal localization by means of creation of a confined layer of water. The water confinement is achieved through the use of a buoyant cellulose sponge thermal insulation. The 3D solar evaporator was kept floating and thermally insulated from the bulk saline water (3.5% wt.) using the cellulose sponge, which has low thermal conductivity. The upper layer of water is connected to the lower bulk water through vertical water transporting macrochannels within the thermal insulation (Fig. 1c). As a result of the neutral buoyancy and macrochannel connection, the self-floating cellulose sponge thermal insulation moves in harmony with the water–air interface, thereby maintaining a stable confined water layer throughout the entire evaporation process. For reference, the direct evaporation rate of the bulk water without the photothermal 3D Ag@rGO was examined. The mass change and evaporation rate of the simulated seawater (3.5% wt.) were presented in Fig. 6c and d, respectively as a function of time under 1 sun illumination. As displayed in Fig. 6c, there was a continuous change in mass over the whole evaporation test, and to account for natural evaporation, evaporation in the dark was conducted under similar conditions.

To evaluate the long-term and stable performance of the 3D evaporator, an interfacial desalination test was carried out for a total duration of 54 h in six cycles of nine hours each. The average flux of water vapor generated in the interfacial desalination setup over the 54-h period was determined to be 1.52 kg m^─2^ h^─1^ under 1 sun solar irradiation and 0.12 kg m^─2^ h^─1^ under dark conditions (Fig. 6d). In contrast, the evaporation rate in the bulk water without the evaporator was found to be 0.233 kg m^─2^ h^─1^ under similar conditions, as opposed to the high vapor generation potential of the solar evaporator. The digital image presented in Fig. 6d (*inset*) and supplementary Fig. 1a and b show the vapor generated by the solar-driven interfacial desalination setup after 30 min of 1 sun solar irradiation. Furthermore, there was no occurrence of salt precipitation on the evaporator body during the 54-h monitoring period in two different tests. This emphasizes the remarkable potential of the evaporator to resist salt, as shown in supplementary Fig. 1d. The surface of the evaporator after the desalination test is comparable to the 3D evaporative surfaces depicted in Supplementary Fig. 1a and c, which were captured at distinct positions prior to commencing the desalination test.

The driving force for the interfacial desalination of the saline water is the surface temperature increase spawned on the evaporator surface. The temperature increase facilitated the fast transfer of water from the cellulose sponge to the evaporative surface via the thermocapillary effect induced by the temperature difference^38^. On account of the low thermal conductivity of the cellulose sponge, the temperature of the bottom of the sponge was at the same temperature as the surface of the water, ensuring that the cellulose sponge effectively suppressed the downward heat leak into the bulk water. In the present study, the 3D Ag@rGO photothermal absorber exhibited broad band solar absorption across the entire solar spectrum, while the interfacial desalination setup depicted in Fig. 2 effectively minimized energy dissipation through radiation to the external environment or conduction to the bulk water. The cellulose sponge served as an insulator to protect heat dissipation and as a water channel to transfer from the bulk to the porous 3D absorber. Incident light absorption in the 3D rGO monolithic structure is enhanced through multiple reflections or scattering of the incident light occurring on the porous surface, with the microcavities playing a significant role in enhancing the absorption of the incident light^15,29,33,65^. The efficient thermal management placed, coupled with the ability of the 3D solar absorber to reuse reflected light via multiple reflections of the incident light owing to its hierarchical architecture, play pivotal roles in attaining the superb photothermal conversion efficiency observed^19,29,56,66^.

The 3D evaporator’s photothermal conversion performance was assessed under 1 sun irradiance, resulting in a surface temperature increase from 27.1 to 54.7 °C. The latent heat of vaporization $\left( {{\text{L}}_{{\text{v}}} } \right)$ at the surface temperature of 54.7 °C is determined to be 2275.2 kJ kg^−1^^67^. A correction factor was applied to account for the absorption of incident solar radiation by the plastic covering of the bottle, with the total solar transmittance of the plastic assumed to be ~ 95% of the total irradiance over the broadband UV–Vis-NIR spectrum^68^. The net solar-driven interfacial evaporation rate was determined to be 1.40 kg m^−2^ h^−1^ after subtracting the evaporation rate in the dark to adjust for natural evaporation. The solar-to-thermal conversion efficiency (η) was found to be 97.54% under one sun solar irradiation for desalinating high salinity (3.5% wt. NaCl) water based on the provided data. In the control experiment conducted without the 3D photothermal material, a water evaporation rate of ~ 0.223 kg m^−2^ h^−1^ was observed.

#### Outdoor solar driven interfacial desalination performance test

**Figure 7:**
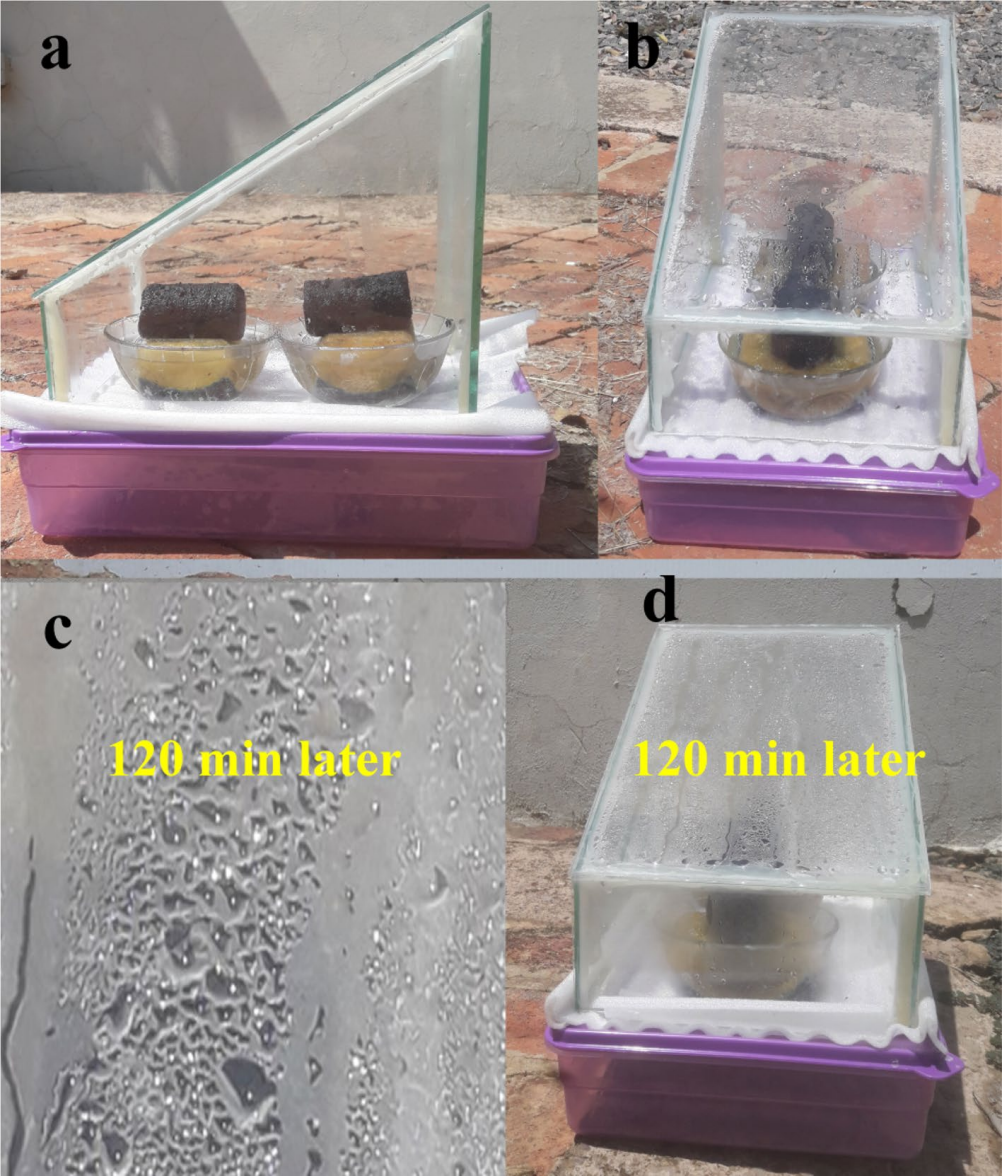
Modified single slope solar still desalination setup consisting of a pair of evaporators positioned on a cellulose sponge, a vapor condenser, and a water collector at the bottom. The transparent glass cover serves the purpose of condensing the fresh water produced and directing it downwards into the vessel through apertures located at the bottom. The side view (**a**), and top view of the desalination setup (**b**); magnified view of the condensate formed on the cover glass 120 min later (**c**), the condensation progress observed on the glass 120 min later (**d**).

The freshwater generation potential of the interfacial desalination setup under real-world conditions was evaluated by placing the setup on a rooftop. In order to evaluate the real word interfacial desalination performance of the 3D Ag@rGO evaporator, a modified single slope solar stills desalination setup was constructed. This setup primarily consists of a pair of evaporators positioned on a cellulose sponge, a vapor condenser, and a water collector at the bottom (as depicted in Fig. 7a–d). An inclined transparent glass cover made of Poly(methyl methacrylate) (PMMA) is utilized to condense and direct the freshwater to the lower part of the vessel. Simulated seawater with a salinity of 3.5 wt.% was used in the study, and the evaluation was conducted from 3:00 am to 5:00 pm, nine hours a day for five consecutive days, from November 22 to November 26, 2023, when there was an average ambient temperature of ~ 30 °C. In the desalination test, the clouds and rainy low light intensity slots were excluded.

**Figure 8:**
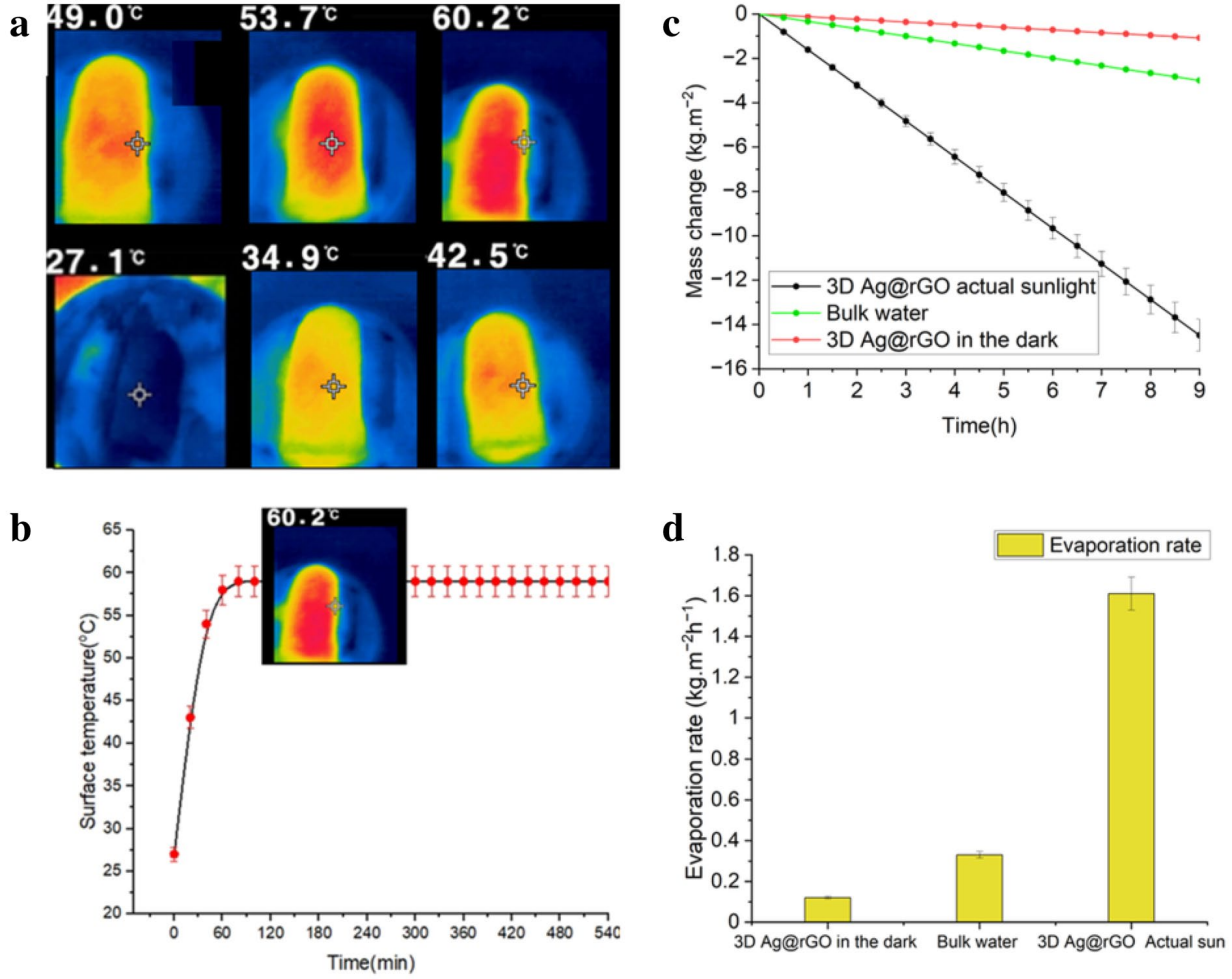
Average surface temperature response profile of the 3D Ag@rGO (**a**); IR thermal images of the 3D Ag@rGO, under natural solar irradiation captured every 20 min from an initial surface temperature of 27.1 °C to ~ 60 ± 3 °C over an hour (**b**); stabilized solar driven interfacial desalination performance of the 3D Ag@rGO in the actual outdoor environment with average mass change of 3.5 wt.% saline water over the 72 h run for nine hours a day for eight consecutive days both under dark condition and under actual solar irradiation as well as under direct bulk heating run in the absence of the evaporator (**c**); average evaporation rate of 3.5 wt.% saline water over the duration of 72 h run for 8 days, nine-hour long each run under dark condition, under direct bulk heating and under natural solar irradiation (**d**).

The surface temperature of the evaporator rose quickly from its initial level of ~ 27.1 °C to ~ 60 ± 3 °C within an hour (Fig. 8a) and eventually stabilized at a value of ~ 60 °C (Fig. 8b), indicating efficient photothermal performance and localization of the heat on the evaporator surface. The surface temperature of the 3D evaporator displayed variability between ~ 57 and  ~ 63 °C an hour later during the bright summer sunlight, thus an average value of ~ 60 °C attained is considered as ${\text{T}}_{2}$ (Fig. 8b). The direct solar evaporation of the bulk water was measured to be 0.333 kg m^─2^ h^─1^. The evaporation rate under the dark condition was determined to be 0.12 kg m^─2^ h^─1^. The interfacial desalination system exhibited change in mass as shown in Fig. 8c, and attained dark excluded freshwater generation rate of 1.51 kg m^─2^.h^─1^, under natural solar irradiation based on the 32.97 cm^2^ evaporative surface area, as illustrated in Fig. 8d. The 3D Ag@rGO evaporator showed no visible salt crystals on the surface and displayed stable performance over the extended desalination study period monitored, suggesting the promising potential of the 3D for real-world application.

In the outdoor field test, the 3D solar evaporator demonstrated a higher surface temperature and produced more freshwater compared to the indoor solar simulation experiment. It is worth noting that the power density of solar irradiance can vary due to factors such as the time of day, season, location, and weather conditions. The field test achieved a higher mass flux rate, which can be attributed to the efficient thermal management system in place that minimizes heat loss and effectively recycles latent heat. With an average solar irradiation density of ~ 750 W m^─2^ over 9 h, reaching up to ~ 1045 W m^─2^ at noon^69^, the interfacial desalination test exhibited 14.58 kg m^─2^ of freshwater extraction potential based on 9 h of desalination test a day and an effective evaporation surface area of 32.97 cm^2^. The evaporation rate remained constant at 1.61 kg m^─2^ h^─1^ throughout the 72-h evaporation test, and no salt crystals formed on the evaporator’s surface. The average daily freshwater yield using natural sunlight is comparable to previously reported yields^70,71^.

**Figure 9:**
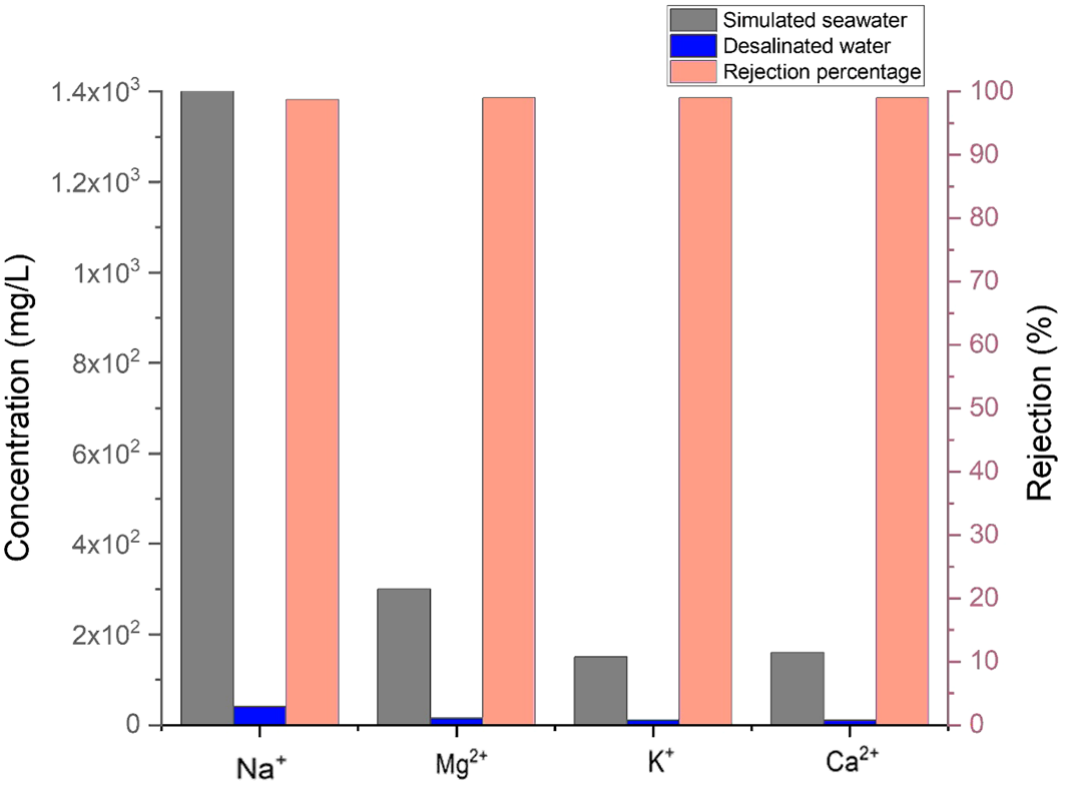
Inductively coupled plasma optical emission spectroscopic analysis of the main salt ions concentrations in both the desalinated water and simulated seawater as well as the percentage of the salt ions rejected in the interfacial desalination process.

An analysis using inductively coupled plasma optical emission spectroscopy (ICP-OES) was conducted on the primary salt ions (3.5 wt% NaCl, KCl, MgSO_4_, CaCl_2_) in both simulated seawater and desalinated water. The results indicated a significant decrease in the concentrations of Na^+^, K^+^, Mg^2+^, and Ca^2+^ ions in the desalinated water, surpassing 99%, as depicted in Fig. 9. These levels comply with the drinking water guidelines established by the World Health Organization (WHO)^20^.

The current photothermal conversion efficiency is comparable to the values reported in the literature under 1 sun solar irradiation^8,38^. The outstanding photothermal efficiency and water vapor generation potential observed can be attributed to the synergistic plasmonic localized surface heating effect of the AgNPs and the waveguide multiple reflections and scattering effect of the microcavities of the porous structure rGO structure, as well as the broadband optical absorption potential^13^. The superb capacity of the 3D Ag@rGO photothermal material to absorb light effectively is evident in both the ultraviolet–visible and near-infrared regions, as depicted in Fig. 5b, ensuring its remarkable photothermal conversion potential. In addition to offering high solar absorption over the broadband solar spectra, the 3D Ag@rGO provides a hierarchical microporous structure for efficient water transport inside the structure. While the all-directional cylindrical Ag@rGO solar evaporator absorbs broadband solar spectra and converts it to heat energy, which is confined to the evaporative surface, the hydrophilic microcavities of the porous structure supply adequate water to the evaporative surface through capillary force and facilitate vapor escape. Hydrophilic cellulose sponge combines excellent thermal insulation and water transport properties^72^.

It can be noted that the photothermal performance exhibited is in direct proportion to the broadband solar absorbance exhibited, as shown in Fig. 5a, b. In addition to the evaporator's efficient solar absorption potential, effective thermal management plays a crucial role in enhancing the photothermal conversion efficiency. Effective thermal management involves minimizing heat loss through conduction to the bulk water, natural convection, and radiation to the environment. To achieve a high solar-to-thermal conversion efficiency, it is essential to boost broadband solar absorbance across the entire solar spectra while simultaneously reducing heat loss to the bulk water and the surrounding environment.

To achieve efficient water vapor generation effective solar absorption, water transfer, and thermal management are critical. Several strategies have been adopted to design and construct novel photothermal materials with high solar absorption and limited thermal emittance and optical reflectance, including defect engineering, material selection, hybridization, and surface engineering^73^. Several factors are taken into consideration when designing solar absorber materials with superior photothermal conversion efficiency. The superb optical property of the 3D Ag@ rGO composite can be attributed to the conjugated π bands in the rGO, that allows for broadband solar absorption of photons across the entire UV–Vis-NIR region. Additionally, the high-density localized plasmonic surface heating effect of the AgNPs contributes to the enhanced photothermal conversion performance of the 3D Ag@rGO, offering the 3D Ag@rGO synergistically improved photothermal conversion performance^26,74^.

The 3D rGO hierarchical structures have residual sp^3^ oxygen-containing carbon chains as junction nodes for covalent bonding between adjacent graphene planes and moderate hydrophilicity for adequate transfer of water to the surface of the evaporator via the thermocapillary effect. As a result of its porosity and compactly interwoven hydrophilic microchannels and microcavities, the hierarchical 3D rGO monolith offers enhanced solar absorption through the waveguide effect and supplies enough water to the evaporative surface through capillary force^31,38^.

The incorporation of metallic nanoparticles into the 3D rGO has been reported to impart multi-functionalities beyond enhanced solar absorption. Despite the remarkable photothermal performance of rGO on account of its sp^2^ conjugated carbon network and abundant delocalized electrons, its photothermal conversion performance suffers from fast recombination of electron–hole pairs^75,76^. The incorporation of AgNPs into the rGO is known to enhance the photothermal performance of the composite through suppression of charge recombination, increasing charge separation performance, and creating an increased reactive surface area^75,77^. Zhu et al.^74^, reported 85% photothermal conversion efficiency of 3D mesoporous plasmonic wood decorated with Au, Ag, and Pd NPs under ten-sun illumination (10 kW m^−2^). The observed efficient solar-to-thermal conversion performance was ascribed to the plasmonic effect of the plasmonic NPs and the waveguide effect of the microchannels in the plasmonic wood. Compared to the traditional solar-driven desalination technologies, where the photothermal material is immersed for bulk volumetric heating, the thermal insulation adopted in the current interfacial evaporation system plays a significant role in effective thermal management and water vapor generation. In the interfacial desalination setup, the cellulose sponge floating on the water surface served as a water channel, preventing direct contact of the photothermal solar evaporator with the bulk water and significantly reducing heat loss into the non-evaporative bulk water.

The major problem with solar-thermal desalination devices is salt precipitation and scaling on the evaporator surface, which heavily compromises the desalination performance as the salt crystals block pores and scatter solar irradiation^78^. The interconnected porous reduced graphene oxide nanosheets with small interlayer spacing are responsible for the promising salt rejection potential observed on the 3D evaporator surface. Through XRD analysis, it was determined that the interlayer spacing of the rGO is approximately 0.350 nm, which is significantly smaller than the diameter of hydrated Na^+^ ion (approximately 0.716 nm)^79,80^. This size difference contributes to the rejection of salt formation on the evaporator body. Additionally, the surface chemistry of the nanopores, along with the charge sieving effect, plays a crucial role in ion exclusion through electrostatic interactions. The high negative surface charge of the rGO nanopores, attributed to the presence of oxygen-containing functional groups on the basal planes and edges, repels negatively charged chloride ions. This further enhances the ion exclusion performance and the overall salt rejection potential of the 3D evaporator^80^. Additionally, the porous 3D structure with highly dense wrinkled morphology on account of the edge-to-edge interconnection of the adjacent graphene nanosheets results in greater salt rejection potential^81^. Thus, the high salt rejection and low salt accumulation sensitivity can be attributed to the sub-nanometric size of the nanochannels and the high charge density of the reduced graphene nanosheets coupled with densely wrinkled porous morphology, conferring excellent ionic selectivity.

**Figure 10:**
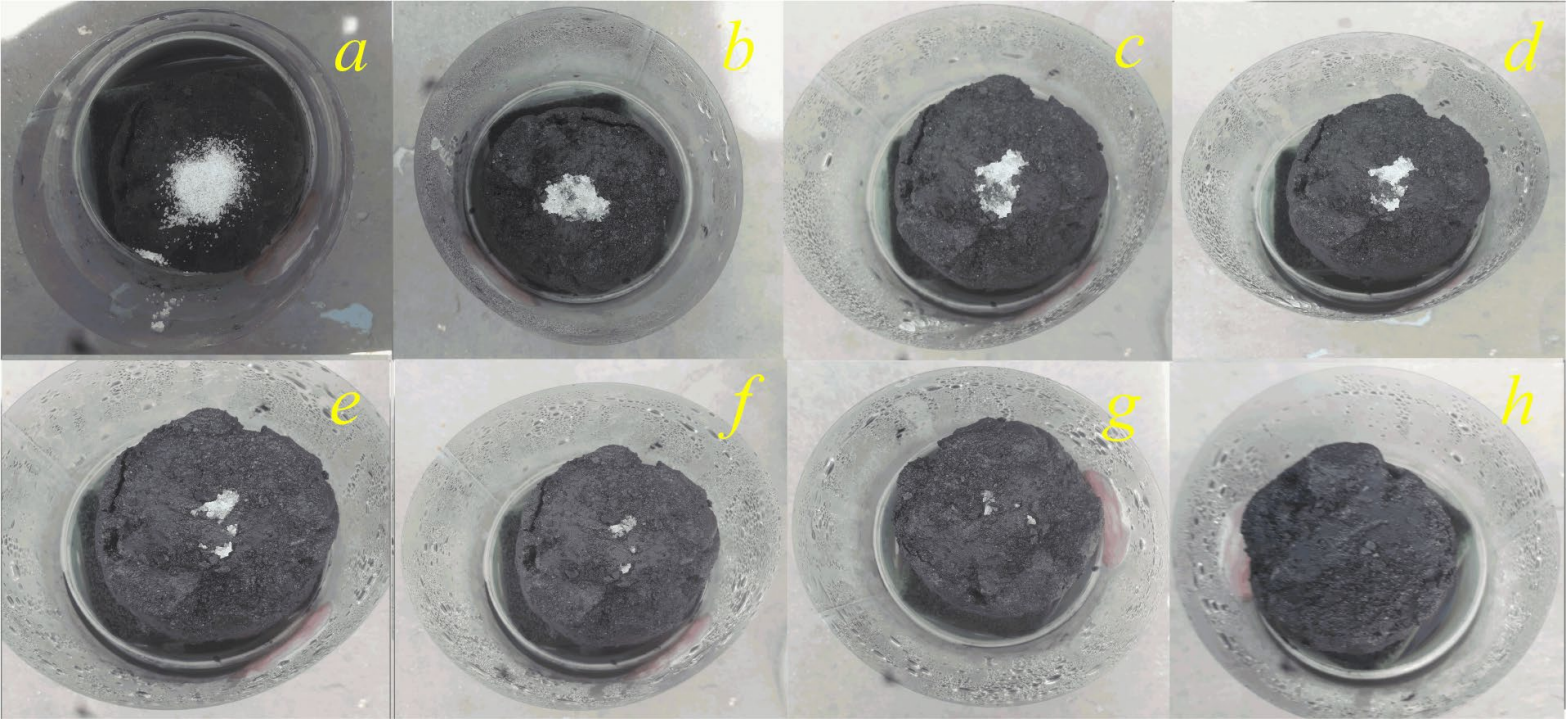
Digital photos captured every hour indicating salt crystals placed on the evaporative surface of the 3D evaporator, which are progressively dissolving from the surface and entering the bulk water beneath.

The salt resistance potential of the 3D Ag@rGO was further investigated by placing 2 g salt crystals on the surface of the evaporator in the interfacial desalination process. Figure 10a–h presents the digital image of the salt crystals’ progressive disappearance, captured every hour, and its complete removal from the evaporative surface of the solar absorber 8 h later.

The high salt rejection potential observed is attributed to the rapid diffusion of the salt ions through the porous water transport channels from high concentration to low concentration between the two ends avoiding salt accumulation and enabling complete dissolution and backwashing of the salt accumulation to the bulk medium^34,78,82^. Hence the high salt rejection potential, can be the combined effect of high porosity and hydrophilicity of the evaporator allowing simultaneous transfer of adequate water to the evaporator surface via capillary force as well as convective and diffusive back wash of concentrated salt back into the bulk water body^34,71,83^. However, the progressive dissolution of the salt crystals followed by concentration gradient driven diffusion or flow of the salt ions into the bulk water can only continue as long as there is a sufficient concentration gradient between the dissolved salt ions on the surface of the evaporator and the bulk water. Additionally, the hydrophilicity of the evaporator surface, which provides enough water to dissolve and wash the salt crystals into the bulk water is the other critical factor which efficiently facilitates the flow of the dissolved salt ions into the bulk water.

## Conclusions

The current study presents the development of a 3D Ag@rGO hierarchical photothermal absorber that exhibits remarkable solar absorption capabilities across the entire solar spectrum. This absorber also demonstrates efficient energy conversion performance without impacting the water transport within its structure. The 3D monolithic structure achieved a high surface temperature, reaching a steady state value of 54.7 °C under 1 sun of irradiation. The 3D Ag@rGO achieved a dark excluded solar-driven water vapor generation rate of 1.40 kg m^−2^ h^−1^ and a photothermal conversion efficiency of 97.54% under 1 sun irradiated interfacial desalination of saline water (3.5% wt. NaCl). The enhanced broadband solar absorption of the 3D rGO can be attributed to the restoration of the disrupted sp^2^ network of graphene and the π–π interactions following the thermal reduction of GO. The high photothermal conversion efficiency and vapor flux are a result of the efficient thermal management setup employed, which incorporates a cellulose sponge at the bottom of the absorber. This sponge provides thermal insulation and minimizes energy dissipation into the bulk water. By localizing the solar energy at the absorber's surface, only the water at the surface is heated, reducing heat loss, and increasing photothermal conversion efficiency. The highly porous surface of the solar absorber, with its abundant micropores, enables rapid water spreading and excellent evaporation properties in the 3D evaporator. The hydrophilic microporous structure of the cellulose sponge, facilitate adequate water supply to the evaporative surface. The current proof-of-concept study on a novel solar-driven 3D Ag@rGO-based interfacial desalination system highlights the significant potential of the 3D solar absorber for real-world broad practical utilization. This work opens up new avenues for further exploration and construction of high-performance novel 3D composite materials with efficient solar-to-heat conversion performance and sustainable freshwater  production from saline water.

### Supplementary Information

Supplementary Figure 1.

## Data Availability

All data generated or analysed during this study are included in this published article and its supplementary information files.
