# Supplementary material for: Design and fabrication of porous three‐dimensional Ag-doped reduced graphene oxide (3D Ag@rGO) composite for interfacial solar desalination
Source: Sci Rep. 2024 Jun 14;14:13793. doi: 10.1038/s41598-024-62987-z (PMC11178800; doi:10.1038/s41598-024-62987-z)
Supplement: Supplementary file 1 — Supplementary Figure 1. [file 41598_2024_62987_MOESM1_ESM.docx]

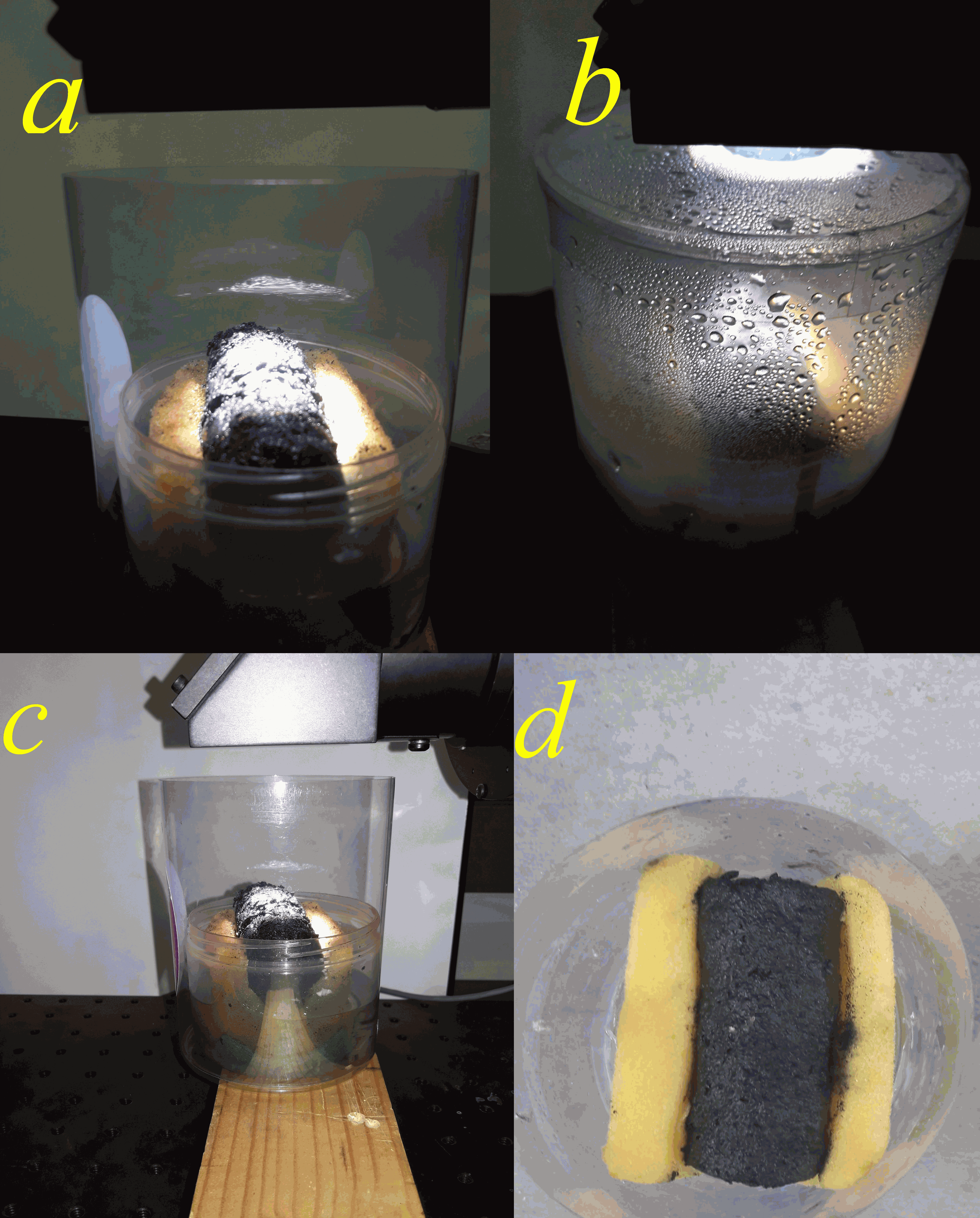


Supplementary Figure 1, Interfacial desalination setups pre (a,c) and post (b) desalination test, along with a photograph of the 3D evaporator post interfacial desalination test (d); a and c display the interfacial desalination setups prior to the commencement of the desalination test, captured at different distances.
